# Nonsymbolic numerosity in sets with illusory-contours exploits a context-sensitive, but contrast-insensitive, visual boundary formation process

**DOI:** 10.3758/s13414-021-02378-y

**Published:** 2021-10-17

**Authors:** Andrea Adriano, Luca Rinaldi, Luisa Girelli

**Affiliations:** 1 Present Address: Department of Psychology, University of Milano-Bicocca, Piazza dell’Ateneo Nuovo 1, Edificio U6, 20126 Milano, Italy; 2 Present Address: Department of Brain and Behavioral Sciences, University of Pavia, Pavia, Italy; 3 Cognitive Psychology Unit, IRCCS Mondino Foundation, Pavia, Italy; 4 NeuroMI, Milan Center for Neuroscience, Milano, Italy

**Keywords:** Visual segmentation, Approximate number system, Illusory contours, Visual illusions

## Abstract

The visual mechanisms underlying approximate numerical representation are still intensely debated because numerosity information is often confounded with continuous sensory cues (e.g., texture density, area, convex hull). However, numerosity is underestimated when a few items are connected by illusory contours (ICs) lines without changing other physical cues, suggesting in turn that numerosity processing may rely on discrete visual input. Yet, in these previous works, ICs were generated by black-on-gray inducers producing an illusory brightness enhancement, which could represent a further continuous sensory confound. To rule out this possibility, we tested participants in a numerical discrimination task in which we manipulated the alignment of 0, 2, or 4 pairs of open/closed inducers and their contrast polarity. In Experiment 1, aligned open inducers had only one polarity (all black or all white) generating ICs lines brighter or darker than the gray background. In Experiment 2, open inducers had always opposite contrast polarity (one black and one white inducer) generating ICs without strong brightness enhancement. In Experiment 3, reverse-contrast inducers were aligned but closed with a line preventing ICs completion. Results showed that underestimation triggered by ICs lines was independent of inducer contrast polarity in both Experiment 1 and Experiment 2, whereas no underestimation was found in Experiment 3. Taken together, these results suggest that mere brightness enhancement is not the primary cause of the numerosity underestimation induced by ICs lines. Rather, a boundary formation mechanism insensitive to contrast polarity may drive the effect, providing further support to the idea that numerosity processing exploits discrete inputs.

**Supplementary Information:**

The online version contains supplementary material available at 10.3758/s13414-021-02378-y.

In a remarkable article published in *Nature* almost 150 years ago, the British economist W. S. Jevons empirically showed that people can rapidly report the approximate number of beans thrown over a table, but error in estimations increased with set size for larger amounts (Jevons, 1871). Nowadays, according to a vast amount of multidisciplinary research, this intuitive elaboration of numerosity (also referred to as “number sense”) is thought to be supported by a dedicated neural network, the so-called approximate number system (ANS; Piazza et al., 2004). The ANS is defined as an evolutionary ancient neural network shared across different animal species (e.g., Agrillo et al., 2009; Agrillo et al., 2012; Brannon & Terrace, 1998; Ditz & Nieder, 2015), and is already active in the early months of life (e.g., Brannon et al., 2004; Xu et al., 2005; Xu & Spelke, 2000). This system would allow living beings to extract approximate numerical information from sensory input stimuli; as such, the ANS may have originally evolved in the animal kingdom as an adaptive mechanism to solve the numerical tasks necessary for survival in the natural environment (e.g., Nieder, 2021).

The main signature of this system is the ratio-based performance obtained in relatively simple behavioral tasks (e.g., Gallistel & Gelman, 2000), in which people have to judge “at a glance” the numerically larger (or the numerically smaller) between two collections of items. This effect is indexed by a decrease of the probability to successfully discriminate two numerosities as their ratio approaches 1 (e.g., smaller numerosity/larger numerosity), in accordance with the notion that nonsymbolic numerosity is internally encoded, like other physical magnitudes (e.g., weight, duration), following Weber’s law (e.g., Whalen et al., 1999).

Another striking behavioral evidence supporting the existence of a dedicated mechanism for nonsymbolic numerical perception is that numerosity information is subjected to adaptation, as are many other primary sensory visual features (e.g., color, speed). Sensory adaptation of selective visual features has been generally used as a psychophysical method to test the existence of neurons encoding a specific feature (Thompson & Burr, 2009). For instance, Burr and Ross (2008) showed that exposure to a large array of objects strongly decreased the perceived numerosity of a subsequently presented array to the region that had been adapted and, vice versa, exposure to a smaller array of dots increased the perceived numerosity of the subsequent array. Hence, in line with this evidence, it has been suggested that numerosity is a primary sensory attribute (or *qualia*; e.g., a dozen strawberries look “twelvish,” just as they look “reddish”; Burr & Ross, 2008). Furthermore, recent studies have shown that numerosity adaptation generalizes across sensory modalities and formats (Anobile et al., 2016; Arrighi et al., 2014). In line with these psychophysical findings, neuroimaging and single-cell recording studies have found neurons selectively tuned to a preferred numerosity in both monkey and human posterior parietal cortex (e.g., Castelli et al., 2006; Harvey et al., 2013; Nieder & Miller, 2004; Piazza et al., 2004), although recent brain imaging and electrophysiological studies suggest that raw numerically related activity in the visual domain might be already detectable at the early stages of processing in the occipital cortex (DeWind et al., 2019; Fornaciai et al., 2017; Fornaciai & Park, 2018; Park et al., 2015; Van Rinsveld et al., 2020).

However, the exact visual features that are employed by the ANS to extract numerical information are still a matter of intense debate. Main computational, psychophysical, and neuroimaging studies (e.g., Burr & Ross, 2008; Dehaene & Changeux, 1993; Grossberg & Repin, 2003; Piazza et al., 2004; Stoianov & Zorzi, 2012; Verguts & Fias, 2004) suggest that numerosity is directly extracted from the retinal input through a primitive visual segmentation mechanism, independently from the position, the size, or the shape of the items composing the visual collection (Dehaene & Changeux, 1993). Generally, computational models suggest a normalization stage in which nonnumerical features are filtered from the sensory input before extracting the numerical information (e.g., Dehaene & Changeux, 1993; Stoianov & Zorzi, 2012). By contrast, several recent studies have shown that the manipulation of physical continuous features confounded in the visual stimuli, such as the density, the item size, the shape, and extent of the convex hull of the collection can affect numerical estimation (e.g., Allik & Tuulmets, 1991; Chakravarthi & Bertamini, 2020; Dakin et al., 2011; Gebuis & Reynvoet, 2012a, 2012b; Hurewitz et al., 2006; Katzin et al., 2020). These theories maintain that numerical representation would primarily rely on continuous features, and not directly on segmented items (e.g., Gebuis & Reynvoet, 2012a, 2012b).

Disentangling the contribution of discrete information (e.g., the perceived number of segmented entities in the set or numerosity) from raw continuous visual features confounded in the stimulus (e.g., spatial frequencies, luminance, density, etc.) is thus critical to examine which visual mechanisms are exploited by the ANS to reach an approximate numerical representation. This is not a trivial problem, because numerosity information is intrinsically confounded with its continuous cues, so that when we manipulate the numerosity of the set we also manipulate several physical cues in the image. Indeed, it seems almost impossible to generate two numerically different ensembles with the same exact amount of continuous features (Gebuis et al., 2016). This has led some authors to propose that numerosity processing can be indirectly accomplished by exploiting more salient continuous cues (e.g., convex hull or the virtual elastic enclosing the items) correlated with numerosity starting from early development (Leibovich et al., 2017).

Recently, visual illusions have been successfully employed to understand which visual features represent the building blocks of numerosity perception, because they can be used to selectively manipulate particular features of numerical sets without altering other physical features in the image (e.g., Dormal et al., 2018; Pecunioso et al., 2020; Picon et al., 2019). For instance, the so-called connectedness illusion has been adopted to manipulate the perceived segmentation (or grouping strength) of the items in the set, keeping constant the low-level features across connectedness levels (Franconeri et al., 2009; He et al., 2009). In these studies, irrelevant lines were used to connect and manipulate the number of dot pairs. This manipulation proportionally reduced the perceived numerosity, likely because the visual system was forced to process two dots as a single unified perceptual object, as suggested by the grouping principle of element connectedness (Palmer & Rock, 1994), representing the input units of visual numerical computation. These findings were also recently replicated with grouping manipulations in which Kanizsa-like illusory contour (IC) lines were used instead of actual physical lines, as the latter may obscure the task-relevant items (e.g., Adriano, Rinaldi, & Girelli, 2021; Kirjakovski & Matsumoto, 2016). ICs are visual experiences of objects whose edges are not defined by physical luminance discontinuities with the background (Nieder, 2002; Wagemans et al., 2012), and thus are well suited to replace the physical lines to simulate connections. In short, studies manipulating either the real or the illusory connecting lines strongly suggest that nonsymbolic numerosity would be extracted from discrete segmented objects rather than from raw low-level features of an unsegmented scene. However, in studies manipulating the IC connections (Adriano, Rinaldi, et al.,  2021; Kirjakovski & Matsumoto, 2016), the illusory lines generated by the inducers were accompanied by a subjective brightness enhancement (e.g., since inducers were darker than the background), with this potentially representing a further “perceived” continuous confound occurring when illusory lines in the sets were increased. The perceived amount of continuous cues in numerical stimuli (rather than mere physical information), as manipulated with classic size illusions, may indeed affect numerical tasks (Dormal et al., 2018; Picon et al., 2019). Furthermore, it has been shown that pupillary diameter may decrease with the illusory brightness enhancement induced by classic Kanizsa illusion and similar brightness illusions (Laeng & Endestad, 2012; Zavagno et al., 2017), which may reduce the light information sampled by the eyes and the visual input, in turn explaining the underestimation effect. Indeed, according to an influential neural theory of vision, ICs emerge as the result of the synergy between two separated but complementary streams in early visual cortex (also known as form-and-color-and-depth model, or FACADE; Grossberg, 2014): the boundary completion system and the surface filling-in system. Consequently, there is often a *subjective* or *perceived* change in brightness that human observers perceive at such illusory contours, but the two processes are governed by two complementary mechanisms. According to this model, one important characteristic separating the two systems is that boundary completion pools across opposite contrast polarities, and thus occurs in a manner that is *insensitive* to contrast polarity of inducers, whereas surface filling in does not pool opposite contrast polarities and is *sensitive* to contrast polarity creating percepts of brightness and color. This “two-systems” division is reinforced by psychophysical studies showing that observers did not perceive strong subjective brightness difference between the illusory surface and the (gray) background when inducing elements had opposite contrast signs (e.g., two white and two black inducers, as in the reverse-contrast Kanizsa square illusion). That is, the perceived brightness of the illusory surface may strongly diminish, or even disappear, when the inducing elements have opposite contrast signs (Grossberg, 2014). In this case, the local signals of differential brightness generated by the individual inducer features should cancel each other out: the brightness induction due to the black-to-grayPac-Man inducers should balance the darkness induction due to the white-to-grayPac-Man inducers. Consequently, no global representation of a brightness difference can be extracted from the stimulus. By contrast, the illusory boundary processing may not be affected by variations in the contrast polarity of the inducing elements since the illusory square is still perceived by the observers when inducers have an opposite contrast sign (e.g., Dresp et al., 1996; Grossberg, 2014; Matthews & Welch, 1997).

Taking advantage of these studies, the current work aimed to disentangle which of the two processes (i.e., boundary completion or surface filling in) actually drive the underestimation effect triggered by Kanizsa-like IC lines (Kirjakovski & Matsumoto, 2016). Hence, we carried out three experiments in which we modulated the number of aligned inducers triggering ICs (zero, two, or four connecting lines) as a function of the contrast polarity (e.g., positive or negative with respect to the background) of the inducing elements. In Experiment 1, inducer pairs triggering the ICs were formed by light-to-gray open inducers (all white) or dark-to-gray open inducers only (all black). In Experiment 2, inducer pairs triggering the ICs had always opposite contrast polarity (one black and one white) compared with the background. In Experiment 3, to exclude further confounds due to item orientation statistics, aligned inducers had opposite contrast polarity, but were closed with a thin line.

If the underestimation of test stimuli is merely due to the perceived brightness enhancement (black inducers triggering ICs brighter than ground), and hence to the surface fill-in system, the effect should be reversed when ICs are darker than ground (white inducers; Experiment 1) and, crucially, no underestimation should be found when inducers of opposite contrast polarity (which suppresses brightness enhancement) were aligned triggering the ICs (Experiment 2). On the other hand, if the underestimation effect will be preserved despite inducers with different contrast polarity (Experiment 1) or simultaneous opposite contrast polarity (Experiment 2) are aligned to create IC connections, we should conclude that this effect is not related to the IC brightness itself. Finally, we predict that in Experiment 3 no underestimation should be found when IC formation was prevented. In such a scenario, the boundary completion system would be the ideal candidate for explaining the observed underestimation effect (Grossberg, 2014).

## Experiment 1: Single contrast polarity open inducers

In Experiment 1, we tested whether the underestimation effect reported in previous studies may depend on the *perceived* change in brightness of the generated illusory surfaces. We manipulated the number of pairs of aligned inducers or ICs (zero, two, or four) and the direction of inducers contrast (positive or negative) compared with the background (e.g., inducers were drawn only in black or white over a middle gray background). Previous studies (Adriano et al., 2021; Kirjakovski & Matsumoto, 2016) employed “brighter” ICs only (generated by black inducers on a gray background), causing a rightward shift of the psychometric functions (e.g., increasing PSE) when ICs were increased. We predicted that if this pattern is merely due to perceived brightness, its direction should be reversed (e.g., leftward shift, or decreasing PSE), or at least differently modulated, when darker ICs (generated by the white inducers) were presented, resulting in a significant interaction between the two independent variables (i.e., numbers of ICs and color of the inducers). Otherwise, a null interaction between IC number and color of the inducers should suggest that numerosity underestimation driven by ICs does not depend on the illusory brightness itself (e.g., no difference in underestimation between black or white inducers).

### Materials and methods

#### Participants

Because of the coronavirus pandemic restrictions in Italy, the participants were recruited through Pavlovia, a repository and launch platform allowing online PsychoPy experiments (www.pavlovia.org). A sample of 28 participants (21 females, seven males) took part in the study.^1^ The mean age was 28.5 years (*SD* = 7.12). Handedness was assessed by asking participants which hand they typically used for writing. A total of 23 subjects were classified as right-handed. All participants had normal or correct-to-normal vision and were naïve about the purpose of the experiment. Each subject signed an online informed consent document before the experiment began, and the study was conducted in accordance with the Declaration of Helsinki. The study was approved by the Local Ethical Committee (protocol N° RM-2020-230).

#### Stimuli

The stimuli set were generated off-line by a custom Python/PsychoPy script and projected by means of an online PsychoPy routine (Peirce, 2007). Stimuli were constructed with the same specifications as in Adriano, Rinaldi, et al. (2021), but with the suited changes to manipulate the contrast polarity of inducing elements (black or white). The whole experimental set was composed of 168 test stimuli (56 random spatial patterns cloned across the three levels of connection, half drawn with black inducers and half with white inducers) and 168 reference stimuli (56 random spatial patterns, 28 with all black inducers and 28 with all white inducers, repeated within the three levels of connection) generated off-line.

The reference stimuli contained a constant number of items (*N* = 12) and were composed by 12 “Pac-Man” like items (diameter = 20 pixels; notch width = 4 pixels; notch length = 10 pixels, measured from the center) spatially scattered and randomly rotated across 360° to avoid collinearities and the pop-out of ICs. The test patterns contained a variable number of items (from 9 to 15 “Pac-Man” like items). We generated a first set of 56 test stimuli for the zero-ICs condition (a total of eight random visual patterns were generated for each of the seven numerosity values), and we coupled them to the 56 reference patterns. In each stimulus of the original zero-ICs set, the distance between the “Pac-Man” items that could prompt the required number of ICs for the other two connectedness conditions was randomly chosen among four possible values (center-to-center distance = 22, 25, 28, and 31 pixels). Reference stimuli were constructed with the same spatial constraints of items in the original test patterns of zero-ICs condition. The inclusion of sets with zero ICs served only as a baseline condition for calculating the PSE in case of no connections (this is indeed necessary to compare the other two conditions—e.g., two ICs or four ICs).

**Fig. 1:**
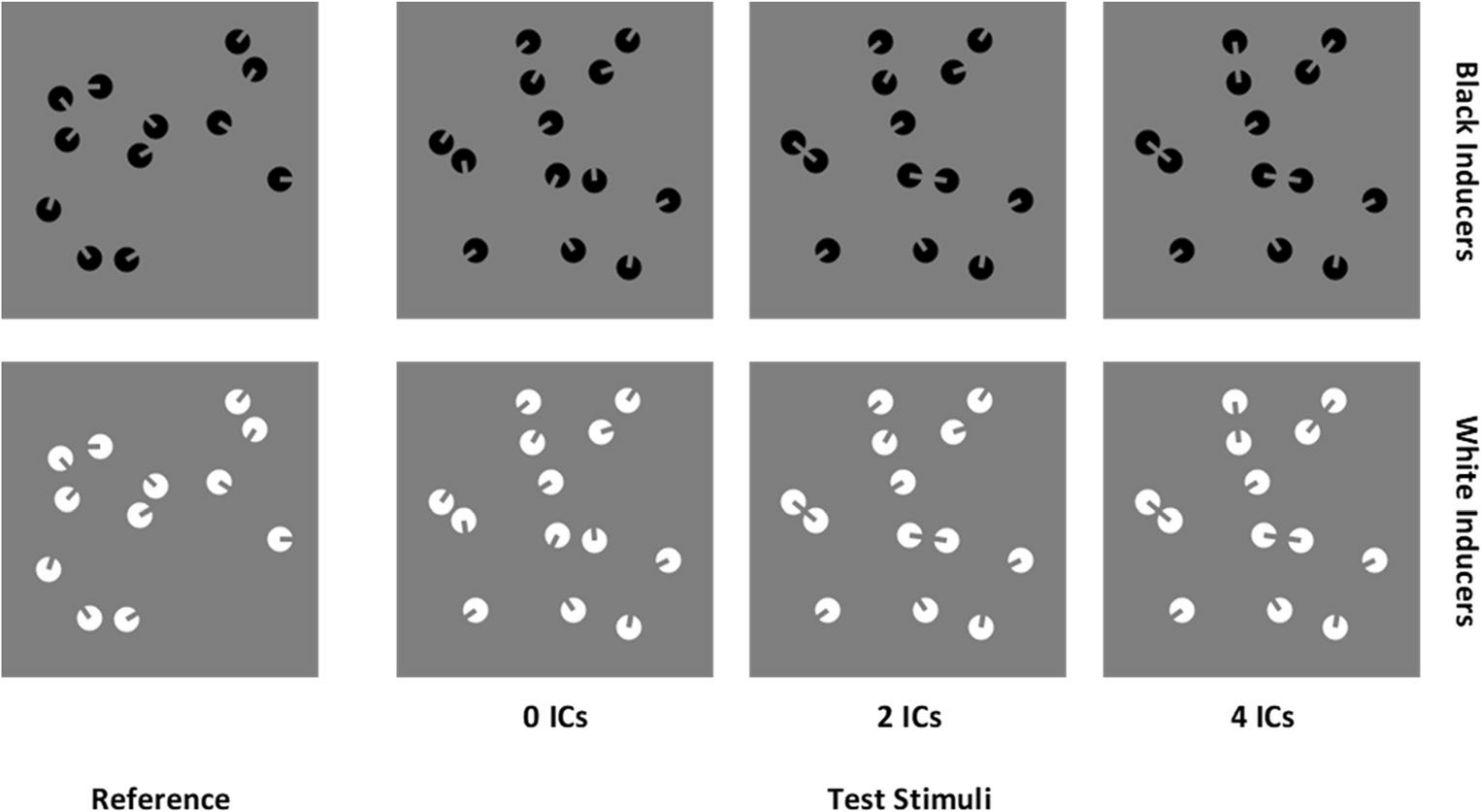
An example of stimuli pairs used in Experiment 1. The reference stimulus was always composed of 12 items. Test patterns varied from 9 to 15 items and contained zero ICs, two ICs, or four ICs. All the test stimuli had the same convex hull, density, and total surface across the conditions. Half of the stimuli were drawn with white inducers, and the other half with black inducers

To keep constant spatial profiles of test sets across the levels of connectedness (i.e., thus controlling for continuous variables such as luminance, density, convex hull, etc.), each different test pattern for each numerosity in the zero-ICs condition was cloned among the different levels of connection for two-ICs and four-ICs test stimuli. Thus, we kept constant the spatial location of all the single items in a given pattern from the zero-ICs set, but a subset of “Pac-Man” items was appropriately rotated and aligned to prompt the required number of ICs for the other test conditions. In this way, the 56 different reference patterns were associated with the same spatial pattern of test stimuli across the levels of connectedness. In sum, a given spatial pattern in test stimuli was cloned across the level of connectedness for each numerosity and was associated with the same reference spatial pattern (see Fig. 1). This ensures that the difference in the PSEs across ICs conditions was not because test patterns were assigned to a different reference spatial pattern across levels (e.g., since the task was a relative magnitude judgment).

All the items in both reference and test stimuli were drawn on a mid-gray background (RGB = 0, 0, 0) and were constrained to be distant at least 20 pixels from the four square edges and to not overlap with each other (minimum center-to-center distance = 22 pixels). Reference and test stimuli were projected within two squared panels (240 × 240 pixels) against a black window (RGB = −1, −1, −1).

#### Procedure

All the participants were individually assessed completing an online PsychoPy routine. The procedure was explained before starting the experiment by providing detailed instructions on the display. No information about the illusions was given. The participants performed a two-alternative forced-choice task (2AFC task) in which they were asked to choose the set containing more dots between two rapidly presented patterns by pressing the corresponding keys on the keyboard.

The experimental phase was preceded by a brief training composed of 24 trials allowing subjects to familiarize themselves with the task. In the training phase, we presented only the condition with 12 versus 9 items (eight trials for each test set with zero, two, or four ICs, half with all black and half with all white inducers). In half of the trials, we presented the numerosity 12 versus nine, while in the other half the opposite order (e.g., 9 vs. 12), with no feedback provided to participants.

**Fig. 2:**
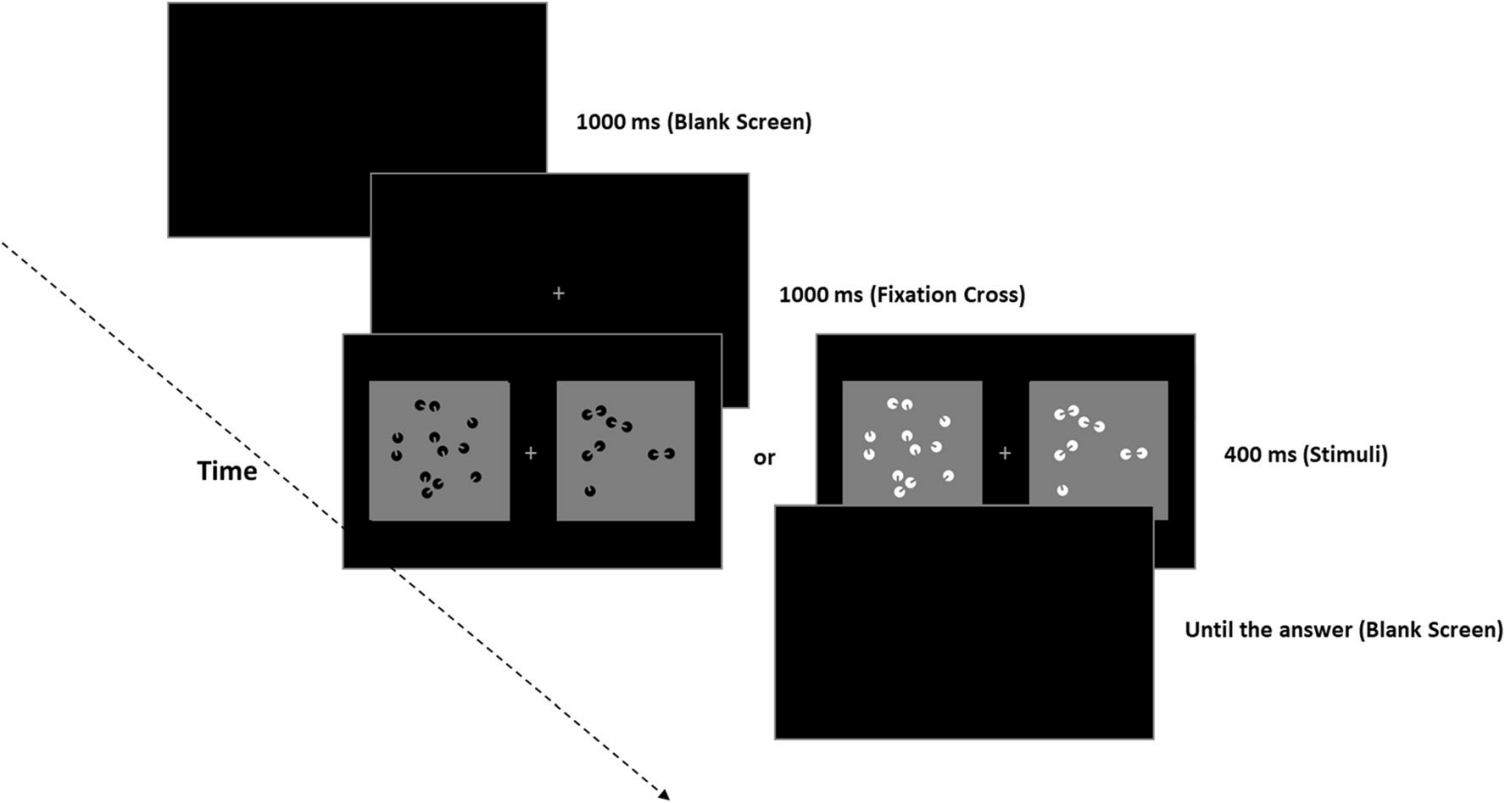
The discrimination task. Subjects had to decide which stimulus was numerically larger by pressing the relative key to specify the left or right stimulus side (F or J key). The side of reference and test pattern was balanced and randomized. In half of the trials, we presented white stimuli (test and reference patterns), while in the other half black stimuli

In each trial of the experimental phase, we always presented a reference set (12 items) and a test stimulus varying from nine to 15 inducers (with zero ICs, two ICs, or four ICs). The position of reference and test stimuli on the screen was completely randomized (reference could appear at the left or right of a fixation cross). Hence, in half of the trials, the smaller numerosity was presented on the left (9 vs. 12; 10 vs. 12), while in the other half, on the right side (e.g., 12 vs. 9; 12 vs. 10). Each trial started with a black background (RGB = −1, −1, −1) lasting 1,000 ms, followed by a fixation cross (font: Times; size: 16 pixels; RGB = 0, 0, 0) projected for 1,000 ms alone, and then two panels appeared at the left or at the right of the fixation cross (72 pixels between the nearest edge of each square panel to the fixation) for an additional 400 ms (see Fig. 2). The side of the reference and test patterns was counterbalanced and randomized across trials. After the stimuli offset, an empty screen (RGB = −1, −1, −1) was presented until the subject’s answer. The subjects could select the stimulus by pressing the appropriate key with their left or right index finger (“F” key for the left stimulus and “J” key for the right stimulus). Response time was not restricted, but we emphasized in the instructions to answer as fast as possible after the stimulus offset. After the practice session, two counterbalanced blocks of 336 randomly ordered trials were presented, for a total of 672 experimental trials (16 trials for each of the 42 conditions), separated by a self-paced pause at the half of the experiment. Hence, all the experimental manipulations were within blocks (50% of the trials with all black or all white inducers) and thus within subjects. The whole experiment lasted around 40–45 min.

#### Data analysis

The data were analyzed with R-Studio (Version 3.6.2; http://www.rstudio.com/) and Jamovi (Version 1.1.5; https://www.jamovi.org) software. Psychometric functions for each condition were generated by fitting Gaussian cumulative distribution functions to the data, and parameters were estimated with a parametric approach based on maximum likelihood method, using Quickpsy package for R (Linares & López-Moliner, 2016). Each of the 42 conditions (i.e., 3 IC conditions × 7 numerosities × 2 inducer colors) contributed with 16 trials to the psychometric function. In order to minimize biases in estimating the psychometric function parameters, we fitted the psychometric curves taking into account the typical lapse in performance (e.g., missing a trial, finger errors) by allowing the value of the guess rate (γ) and lapse rate (λ) parameters to vary in the default range of 0–0.05 (Wichmann & Hill, 2001).

**Fig. 3:**
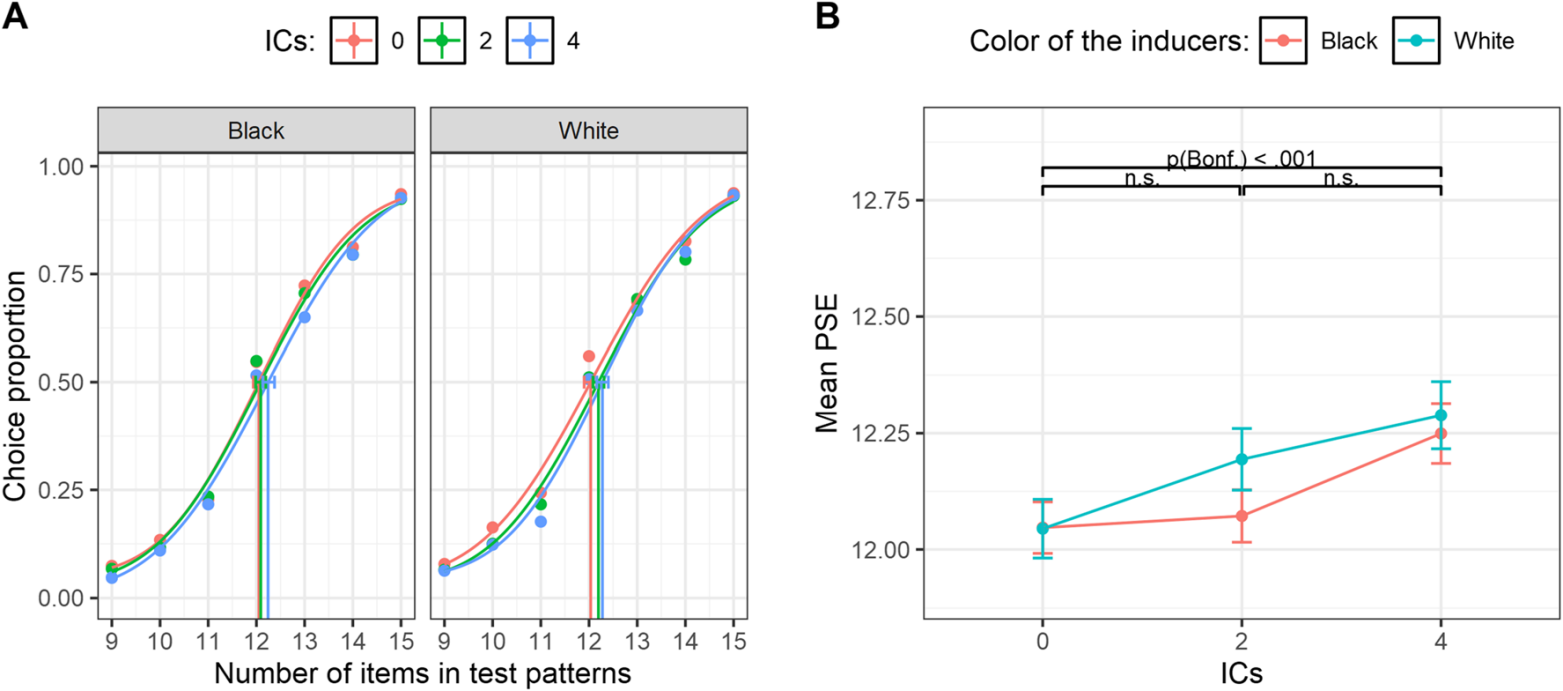
**a** Psychometric functions obtained in Experiment 1 for zero, two, and four ICs as a function of the color of the inducers (black or white), obtained pooling over the aggregate data of all the subjects. The *x*-axis represents the actual number of items in test patterns, whereas the *y*-axis shows the proportion of test patterns that were judged as more numerous than the reference. Vertical lines represent the PSE (0.5 threshold level) for each condition. The error bars represent the bootstrap 95% confidence intervals. **b** The *x*-axis shows the number of ICs as a function of the color of the inducers, and the *y*-axis the mean PSE for each condition in the whole sample. The error bars represent ± 1 *SEM*

To investigate the effect of the manipulations over perceived numerosity, we calculated the point of subjective equality (PSE) for each IC condition and for each inducer color type as a function of the numerosity in test set—that is, the number of dots in test patterns required in order to be subjectively judged as equal to the reference patterns (12 items). The 50% of the chosen test patterns was set as threshold level. In the graphical representation of psychometric curves, the *x*-axis represents the actual number of items in test patterns, whereas the *y*-axis shows the proportion of trials in wich test patterns were judged as more numerous than the reference (see Fig. 3a). Different psychometric curves are plotted depending on the number of aligned inducer pairs (e.g., different colors) in function of the inducer type (black or white).

The 95% confidence intervals of individual PSEs were estimated running 200 bootstrap resampling of the data. Furthermore, as an index of the precision of the numerical discrimination and to confirm that the performance follows Weber’s law, we calculated the coeffienct of variation (CoV; Halberda & Odic, 2014; Whalen et al., 1999), as the ratio between the standard deviation (*SD*) and the PSE of the psychometric functions for each condition.

Two separate two-way repeated-measures analyses of variance (ANOVAs) were performed with the number of ICs (zero, two, or four) and the inducers color (white or black) as within-subjects factors and with the mean PSE or the mean CoV as dependent variables, respectively. The Greenhouse–Geisser epsilon (ε) correction for violation of sphericity was applied when needed, and original *F*, *df* and corrected *p* values were reported. We also  ran Bayesian analyses (see the Supplementary Materials).

### Results

As is shown in the figure plotting the psychometric functions obtained pooling over the aggregate data of the whole sample (Fig. 3a), we found a rightward shift of the psychometric curves when darker or brighter ICs were increased, suggesting a systematic underestimation of the perceived numerosity as we increased the number of connections regardless of the color of the inducers (note that this graph is reported to illustrate the technique, but all subsequent analysis was done with similar functions over individual subjects; see Fig. S1 in the Supplementary Materials for individual data). The analysis of individual PSEs showed a significant main effect of the number of ICs, *F*(2, 54) = 8.06, *p* < .001, η_p_^2^ = .23. That is, the PSEs increased with the number of ICs (Fig. 3b). Such a pattern was confirmed statistically by means of post hoc comparisons (Bonferroni correction), revealing a significant difference between zero ICs and four ICs, *t*(54) = −3.98, *p *< .001, while no significant difference was found between zero ICs and two ICs, *t*(54) = −1.55, *p* = .37, and between two ICs and four ICs, *t*(54) = −2.42, *p* = .056. A polynomial trend analysis showed a significant linear trend only, *t*(54) = 3.98, *p <* .001*.* Furthermore, the main effect of inducer color was not statistically significant, *F*(1, 27) = 1.83, *p* = .187, η_p_^2^ = .06, and crucially no significant interaction between the two factors was found, *F*(2, 54) = .57, ε = .80, *p* = .52, η_p_^2^ = .02, suggesting a similar underestimation with black or white inducers.

In addition to this, the analysis of the CoV of the psychometric functions revealed no significant main effect of number of ICs, *F*(2, 54) = 2.1, ε = .80, *p* = .142, η_p_^2^ = .07, no significant main effect of inducers color, *F*(1, 27) = .43, *p* = .51, η_p_^2^ = .016, and no significant interaction, *F*(2, 54) = 1.59, *p* = .21, η_p_^2^ = .056, suggesting that participants numerical acuity was stable across all the conditions as predicted by the Weber’s law (see Supplementary Materials and Fig. S2). In addition to frequentist analyses, we also ran Bayesian ANOVAs over both the PSEs and the CoVs with the number of ICs and inducer colors as independent variables. These additional analyses confirmed the main results reported here (see Supplementary Materials for more details; see also Table S1 and Table S2).

### Discussion of Experiment 1

The results of Experiment 1 revealed that the color of the inducers did not affect participants’ numerical estimations. Furthermore, the effect of ICs was found to be statistically significant, and, crucially, no interaction was found with the inducer color type, showing a similar increasing pattern in PSE over both color types. This suggests that more items were required in test patterns to be perceived numerically equal to the reference when inducer pairs were aligned, independently of the polarity (or contrast direction) of the inducers and the relative change in surface brightness (brighter or darker than the background). Hence, it is unlikely that the filling-in process might be the source of this effect, indirectly suggesting that the boundary system (which is insensitive to contrast polarity) would drive the underestimation effect. To directly probe this possibility, in Experiment 2, we tested participants with stimuli composed of opposite-contrast inducers, which should strongly reduce the perceived brightness of the generated ICs shapes (Grossberg, 2014).

## Experiment 2: Reverse-contrast polarity open inducers

To corroborate and extend the results of the first experiment, in Experiment 2 we tested whether the underestimation effect is preserved when IC lines were generated by inducers with reverse contrast polarity. If the underestimation effect is merely a by-product of the IC brightness, no underestimation effect should be found when reverse contrast polarity inducers were aligned (e.g., one black and one white), since in this case the perceived difference in luminance of the illusory surface is strongly reduced (e.g., Dresp et al., 1996; Grossberg, 2014; Matthews & Welch, 1997). Otherwise, if the boundary contour system (which is insensitive to contrast polarity of inducers) drives the effect, the underestimation pattern should be preserved even when inducers have opposite contrast polarity.

### Materials and methods

#### Participants

A new sample of 23 participants (14 females) was recruited for the second online study.^2^ The mean age was 30.73 years (*SD* = 7.91). A total of 22 participants were classified as right-handed. All the subjects had correct or corrected-to-normal vision and were naïve to the goal of the study.

#### Stimuli and procedure

**Fig. 4:**
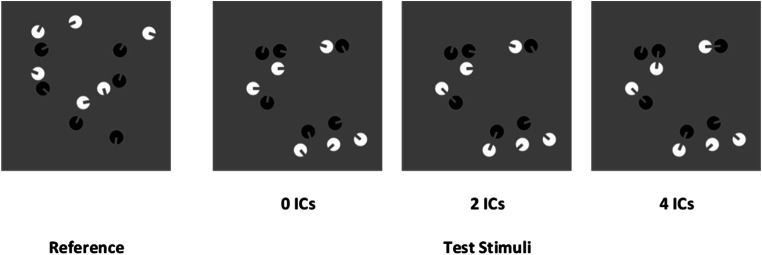
An example of stimuli pairs used in Experiment 2. The reference set was always composed of 12 items (half black and half white inducers). Test patterns varied from 9 to 15 items and contained zero ICs, two ICs, or four ICs. All the test stimuli had the same convex hull, density, and total surface across the levels of connectedness (as in the examples depicted)

The stimuli were generated as in Experiment 1, but the inducers had mixed polarity. The reference patterns were composed by 12 “Pac-Man” like items but half of the items were drawn in white (RGB = 1, 1, 1) and the other half in black (RGB = −1, −1, −1). As in the first experiment, test patterns contained a variable numerosity (from 9 to 15 “Pac-Man” like items), but those containing an even numerosity (10, 12, 14) were constructed with an equal number of black and white inducers on a mid-gray background (e.g., Kogo et al., 2014), whereas test stimuli with odd numerosity (9, 11, 13, 15) were counterbalanced, containing one free inducer in excess drawn in black in half of the patterns (four random visual patterns) and drawn in white in the other half (four random visual patterns). Note that aligned inducers forming the ICs in test stimuli had always one black and one white inducer (see Fig. 4). All the items were drawn on a mid-gray background (RGB = 0, 0, 0).

### Results

Two separate one-way repeated-measures ANOVAs were carried out with either the PSE or the CoV of the psychometric functions as dependent variables and the number of ICs (zero, two, or four) as within-subjects variable.

**Fig. 5:**
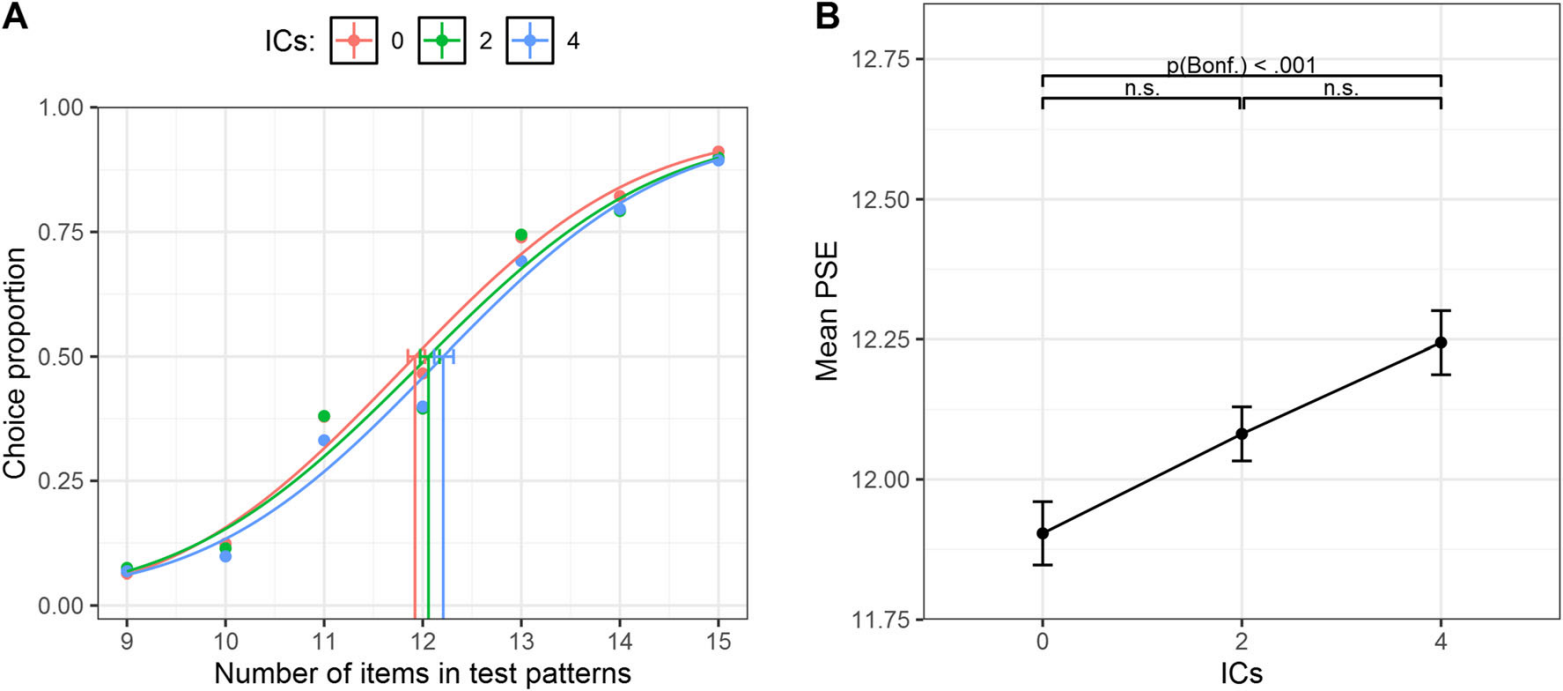
**a** Psychometric functions obtained in Experiment 2 for zero, two, and four ICs, pooling over the aggregate data of all the subjects. The *x*-axis represents the actual number of items in test patterns, whereas the *y*-axis shows the proportion of test patterns that were judged as more numerous than the reference. Vertical lines represent the PSE (0.5 threshold level) for each condition. The error bars represent the bootstrap 95% confidence intervals. **b** The *x*-axis shows the number of ICs and the *y*-axis the mean PSE for each condition in the whole sample. The error bars represent ±1 *SEM*

Visual inspection of the figure plotting the psychometric functions obtained pooling over the aggregate sample data (see Fig. 5a), suggests a rightward shift of the psychometric curves for two and four IC conditions, thus suggesting an underestimation of the perceived numerosity as we increased the number of connections in test stimuli (see Fig. S3 in the Supplementary Materials for individual data). The analysis of individual PSEs showed a significant effect of the number of ICs, *F*(2, 44) =11.14, *p* < .001, η_p_^2^ = .33. That is, the PSEs increased with the number of ICs (see Fig. 5b). Such a pattern, was confirmed statistically by means of post hoc comparisons (Bonferroni correction), revealing a significant difference between zero ICs and four ICs, *t*(44) = −4.72, *p* < .001, while no significant difference was found between zero ICs and two ICs, *t*(44) = −2.46, *p* = .053, and between two ICs and four ICs, *t*(44) = −2.25, *p* = .087. Furthermore, a polynomial trend analysis showed a significant linear trend only, *t*(44) = 4.72, *p <* .001*.*

The analysis of the CoV of the psychometric functions for the three levels of connectedness showed no significant differences across conditions, *F*(2, 44) = 1.58, *p* = .21, η_p_^2^ = .067, suggesting an equal numerical estimation precision across ICs conditions as predicted by the Weber’s law (see Fig. S4 in the Supplementary Materials).

In addition to frequentist analyses, we also ran Bayesian ANOVAs over both the PSEs and the CoVs, with the number of ICs as independent variable. These additional analyses confirmed the main results reported here (see Supplementary Materials for more details; see also Table S3 and Table S4).

### Discussion of Experiment 2

The increase in the PSEs found in Experiment 2 suggests a decrease of perceived numerosity (underestimation) caused by the grouping of few individual items into pairs. Crucially, the results of this experiment corroborate the idea that the numerical underestimation effect triggered by the IC lines is independent from the perceived brightness enhancement that accompanies Kanizsa-like illusory figures. Indeed, numerosity was underestimated (e.g., PSE increased) as the number of IC lines was manipulated, even though the ICs were triggered by opposite contrast polarity inducers that did not produce substantial brightness enhancement (e.g., Grossberg, 2014). This strongly suggests that the numerosity underestimation effect found in previous studies (e.g., Adriano et al., 2021; Kirjakovski & Matsumoto, 2016) was driven by the boundaries completion system (e.g., Grossberg, 2014). Hence, the numerical underestimation effect was actually due to the binding of the inducers into a dumbbell object and cannot be explained by the sensory confounds that brightness enhancement may have involuntary introduced.

However, such findings may be still explained by a general effect due to inducers edge alignment and/or item orientation statistics rather than by the boundary completion of the illusory lines (e.g., DeWind et al., 2020). To exclude this possibility, in Experiment 3, the “Pac-Man” shapes were the same as in Experiment 2, but each inducer was closed with a line to prevent IC formation.

## Experiment 3: Reverse-contrast polarity closed inducers

Previous studies showed that closing the notch of inducers with a thin line strongly reduced the formation of Kanizsa-like ICs and blocked the secondary visual cortex (V2) response (Davis & Driver, 1994; Peterhans & von der Heydt, 1989, 1991; von der Heydt et al., 1984). Here, we predicted that if the results of Experiment 1 and Experiment 2 were due to mere inducer orientation statistics, rather than to the specific completion of the ICs lines, we should find an equal increase in the PSEs when the notch of the closed inducers were spatially aligned as in the previous experiments. A lack of effect would suggest that underestimation effect was specifically driven by the illusory boundary and not by inducers’ orientation statistics (Kirjakovski & Matsumoto, 2016).

### Materials and methods

#### Participants

A new sample of 24 undergraduate and postgraduate students (mean age ± *SD* = 26.87 ± 4.19 years, 17 females, 23 right-handed), with normal or correct-to-normal vision, was recruited for the third online study.^3^ All the subjects were naïve regarding the purpose of the experiment.

#### Stimuli and procedure

**Fig. 6:**
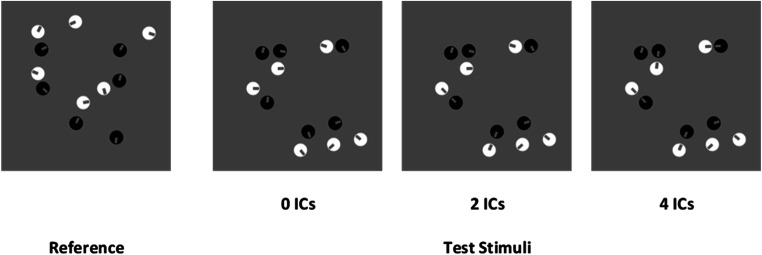
An example of stimuli pairs used in Experiment 3. The reference set was always composed of 12 items (half black and half white inducers). Test patterns varied from 9 to 15 items and contained zero ICs, two ICs, or four ICs. All the test stimuli had the same convex hull, density, and total surface across the levels of connectedness (as in the examples depicted)

The design, stimuli parameters and their generation method as well as the procedure were identical to Experiment 2. The only difference in the visual stimuli patterns was that inducers were closed with a curved line (1 pixels thick), thus completing the overall circular shape of each item. Note that stimuli spatial patterns were cloned from stimuli with open inducers (see Fig. 6). For the sake of clarity, to define the name of the experimental conditions with aligned closed inducers we adopted the same labeling as in Experiment 2 (e.g., zero ICs, two ICs, and four ICs).

### Results

**Fig. 7:**
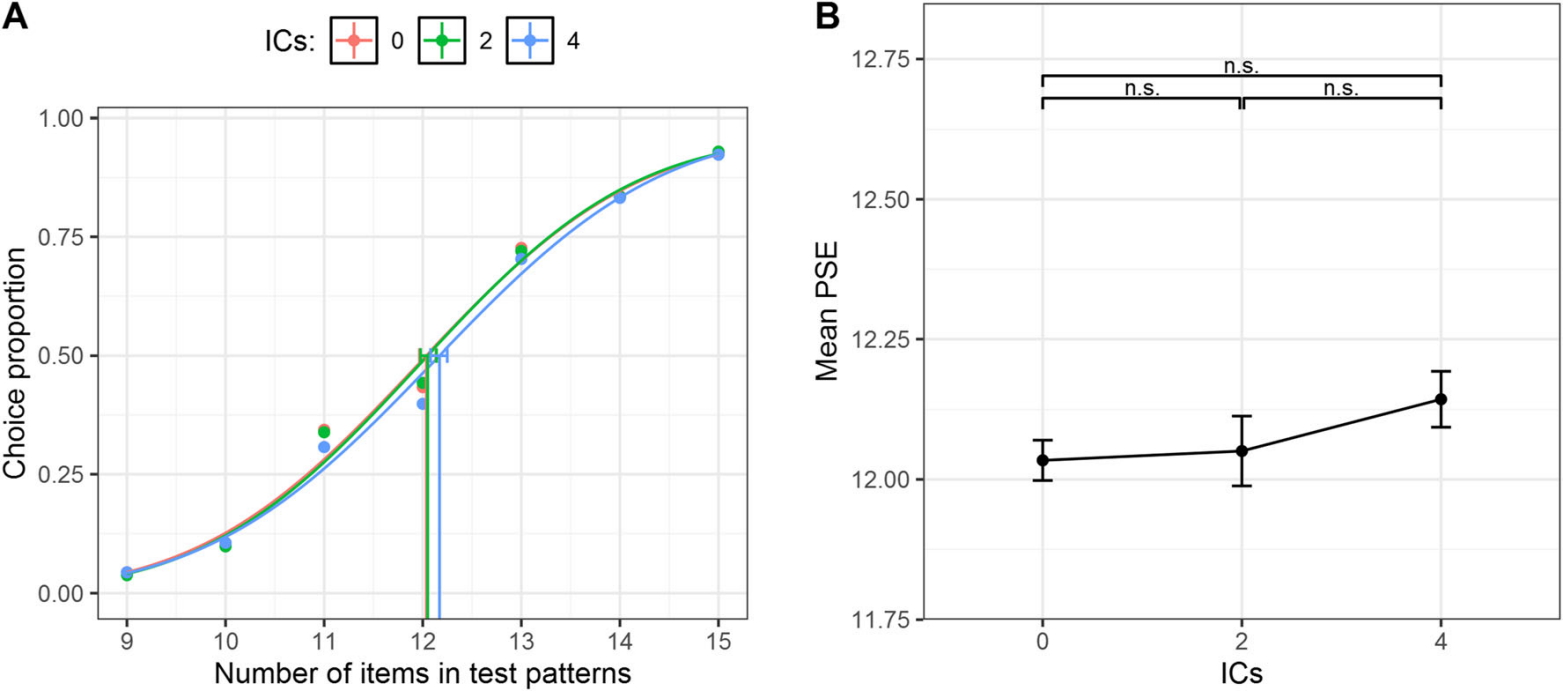
**a** Psychometric functions obtained in Experiment 3 for zero, two, and four ICs, pooling over the aggregate data of all the subjects. The *x*-axis represents the actual number of items in test patterns, whereas the *y*-axis shows the proportion of test patterns that were judged as more numerous than the reference. Vertical lines represent the PSE (0.5 threshold level) for each condition. The error bars represent the bootstrap 95% confidence intervals. **b** The *x*-axis shows the number of ICs and the *y*-axis the mean PSE for each condition in the whole sample. The error bars represent ±1 *SEM*

Data were analyzed as in Experiment 2(see Fig. S5 in the Supplementary Materials for the individual psychometric functions). Two separate one-way repeated-measures ANOVAs were carried out with either the PSE or the CoV of the psychometric functions as dependent variables and the number of ICs (zero, two, or four) as the independent variable. As expected, the analyses showed no significant difference across the conditions, as the number of ICs did not affect the PSEs, *F*(2, 46) = 1.5, *p* = .23, η_p_^2^ = .06 (see Fig. 7a and b). Similarly, no effect of ICs condition was found for the CoV, *F*(2, 46) = .154, *p* = .85, η_p_^2^ = .007 (see Fig. S6), suggesting an equal numerical precision across conditions. Finally, we also ran supplementary Bayesian statistics over both the PSE and the CoV (See Supplementary Materials for more details; see also Table S5 and Table S6), which overall confirmed frequentist analyses.

### Discussion of Experiment 3

The results of Experiment 3 indicate that the mere manipulation of spatial alignment (or orientation) of edges between a few inducers was not sufficient to group the items into a perceptual object producing the underestimation obtained with open inducers (e.g., Davis & Driver, 1994; Kirjakovski & Matsumoto, 2016). Furthermore, these results suggest that a mere difference in item orientation statistics across conditions cannot explain the results of previous experiments (DeWind et al., 2020), replicating prior evidence with only black inducers (e.g., Adriano et al., 2021; Kirjakovski & Matsumoto, 2016). The slight increase in PSE of Experiment 3 could also be in line with previous studies using similar closed inducers (e.g., Chen et al., 2018), but adopting a dot-localization task. Indeed, Chen et al. (2018) found that closing the notch does not completely suppress the effect of surface completion, especially when only a tiny closing line is used. It is therefore possible that, in Experiment 3, closed inducers still triggered a weak grouping (as showed by the slightly increase in PSE; see Fig. 7b), since in the periphery visual acuity decreased and the 1-px closing lines sometimes can be misperceived or not be sufficient to completely suppress the surface completion. Note that we recently tested two different types of closing-line size (1 px and 4 px), and in both cases no effect of IC alignment was found (Adriano, Rinaldi, et al., 2021).

However, and crucially, the amount of rotation of the inducers and the overall spatial position of the items were exactly the same across Experiment 2 and Experiment 3. Hence, if the change in PSE in Experiment 2 was simply due to mere inducer orientation statistics, a strong effect of item rotation should also have been found in Experiment 3. Yet we found only a small (nonsignificant) effect of inducer alignment in Experiment 3(see also Adriano, Rinaldi, et al., 2021; Kirjakovski & Matsumoto, 2016), which could be compatible with the findings of Chen et al. (2018), but not with the idea that orientation statistics drive the increase in PSE per se. These findings further corroborate the idea that the underestimation effect found in Experiment 1 and Experiment 2 is specifically due to the *strong* binding of the single items in the pairs driven by the (modal) IC connecting lines, generated by a boundary system insensitive to the contrast polarity of the inducers, rather than to mere inducers orientation statistics.

## General discussion

Recent studies in numerical cognition have employed visual illusions as a powerful tool to reveal the exact mechanisms underlying visual perception of numerosity (Adriano et al. 2021; Dormal et al., 2018; Franconeri et al., 2009; He et al., 2009; Picon et al., 2019). Specifically, Kanizsa-like illusory contours have been used to precisely manipulate connectedness level (or grouping strength) of the individual dots (Adriano et al., 2021; Kirjakovski & Matsumoto, 2016) without changing main low-level features in the scene (e.g., density, occupancy, total area, convex hull, etc.). Overall, this line of research indicates that a rapid visual scene segmentation mechanism might be at the root of visual numerosity extraction (e.g., Burr & Ross, 2008; Dehaene & Changeux, 1993; Grossberg & Repin, 2003; Piazza et al., 2004; Stoianov & Zorzi, 2012; Verguts & Fias, 2004), rather than mere continuous cues and/or texture statistics (e.g., Dakin et al., 2011; Gebuis et al., 2016; Leibovich et al., 2017). However, such illusory figures may have involuntary introduced further uncontrolled visual continuous confounds, such as the perceived brightness in visual scene. Hence, the observed underestimation reported by previous studies might be potentially explained by uncontrolled continuous cues, and/or as a reduced light sampling of visual input, rather than by the grouping itself of the single inducers into discrete segmented objects.

To shed light on this issue, here, we manipulated the perceived brightness level of the illusory contours. Our results clearly indicate that underestimation triggered by ICs lines did not depend on the perceived change in brightness of the illusory surface per se. Rather, the fact that the underestimation is preserved when inducers had opposite contrast polarity, regardless of the color of the inducers (all black or all white) as in Experiment 1, or one black and one white as in Experiment 2, strongly suggests that a mechanism insensitive to contrast direction may drive the numerical underestimation. Finally, in Experiment 3, we found that the underestimation effect was specifically due to the illusory boundary formation and not to other image statistics (e.g., items orientation).

Beside this, we believe that the different change in PSE in Experiment 1 and Experiment 2(as compared with Experiment 3) suggests that our findings cannot be explained by long-term memory effects. If participants memorized the overall spatial pattern or even the single position of each item in test or reference stimulus, no difference in perceived numerosity (PSE) should be found across ICs conditions in Experiment 1 and Experiment 2, since stimuli pairs had all the same patterns across ICs conditions. Finally, we totally exclude an effect of memory because closed inducers and open inducers (Experiment 2 vs. Experiment 3), were also cloned across experiments. In Experiment 3, we presented the same trials and stimuli pairs (yet, in random order) with exactly the same visual patterns and item position of Experiment 2(cf. Fig. 4 vs. Fig. 6). Because the pattern of PSE was very different across experiments but, overall, stimuli spatial patterns were equals, we are confident that long-term memory effects cannot account for the overall patterns of results within or between experiments

According to the FACADE model, ICs are encoded by two interacting but complementary streams in early visual cortex (e.g., Grossberg, 2014). In particular, as supported by psychophysical and computational data, ICs are generated by a boundary mechanism *insensitive* to opposite contrast polarity and by a filling-in mechanism *sensitive* to the direction of contrast (Dresp et al., 1996; Grossberg, 2014; Matthews & Welch, 1997). This model might explain why when the notches of the two single inducers are collinear (independently of their polarity), they instantiate the connecting illusory line so that the Pac-Man shapes are perceived as forming one dumbbell-like object, as the grouping by element connectedness effect would suggest (e.g., Palmer & Rock, 1994). That is, a neural mechanism in visual cortex should trigger the IC-connecting line taking as input the two single separated inducers, thus forcing the two inducers to be processed as a unified single connected object. Neural models suggest that this operation is carried out in neurons with properties similar to a logical AND gate, called bipole grouping cells. Indeed, neurons with similar features, have been found in monkey V2 cortex (Baumann et al., 1997; von der Heydt et al., 1984), and their properties have also been confirmed by later psychophysical work (Field et al., 1993; Shipley & Kellman, 1992). Bipole cells can complete boundaries in response to colinear inducers with the same relative contrast and between inducers with opposite relative contrasts with respect to the background, receiving their inputs from complex cells in layer 2/3 of cortical area V1. Complex cells, in turn, pool inputs from simple cells in layer 4 of V1 that have the same preferences for position and orientation, but opposite contrast polarities (e.g., Dresp & Grossberg, 2016). Later neural models and psychophysical data (Kogo et al., 2010) have highlighted additional specific features and constraints allowing the emergence of the ICs (see also Spehar, 2000).

However, we pinpoint that in the current work the “context” in which we use this classic Kanizsa-like illusion revealed a systematic *underestimation* of numerosity, since the grouped inducers were perceived as belonging to a “whole” single object. Indeed, the illusion used in the current study is a combination of two illusions tapping onto different but strictly related aspects of perceptual organization that have been mostly investigated separately: the grouping principle of element connectedness defining the entry-level unit of visual perception (e.g., Palmer & Rock, 1994) and the specific rules governing the emergence of the classic Kanizsa illusion as well as the filling-in process (e.g., Grossberg, 2014). While many psychophysical studies have investigated the rules governing the filling-in and boundary completion in ICs manipulating inducer features such as contrast and shape (Dresp et al., 1996; Grossberg, 2014; Kogo et al., 2010; Matthews & Welch, 1997; Spehar, 2000), little attention has been directed to the unifying effect of this emergent illusory shape over the perceptual organization of the overall figure, formed in this case by the two inducers *and* the connecting illusory line (e.g., hence forming a dumbbell-like object). Therefore, in our work the IC generated by the two inducers was totally task irrelevant, since subjects were instructed to estimate the number of inducer shapes. This striking combination of illusions (e.g., element connectedness driven by ICs) with the specific task-context used, in which inducers were the to-be-counted items, may reveal a form of grouping by element connectedness occurring even though two physically separated surfaces are illusorily connected. Recently, Roelfsema and Houtkamp (2011) suggested a neural model suited to explain the effect of physical connectedness that can also accommodate the case of illusory connectedness and other grouping rules. In particular, when inducer elements are not directly connected by a physical line but by other grouping rules such as good continuation, the model assumes a spread of enhanced neural activity through horizontal connections between neurons tuned to well-aligned contour elements (Field et al., 1993; Grossberg & Raizada, 2000), corresponding to the spread of object-based attention at the psychological level.

In sum, the use of the ICs in the context of a numerical task, in which inducers were task relevant items, suggests that the illusory boundary triggers an overall organization of the inducers into a global shape. This “hidden” grouping effect might have been overlooked in classic research about ICs since inducer features were often manipulated to study the factors underlying the grouping of the aligned inducer-edges triggering the emergence of (task-relevant) illusory shape and brightness. Thus, the task-context itself was not favorable to capture this (context-dependent) grouping mechanism generated by the ICs over the hierarchical organization of the numerosity input units. Indeed, as we emphasize, grouping is not working only at the level of the inducers collinear edges generating the ICs line, but once this line is triggered, the inducers are likely grouped in a coherent whole configurational object (as if the line were a complete physical line), and this whole object is then selected as a single input unit for numerosity computation. Our results are also in line with previous studies in which connectedness manipulation was used. For instance, recent studies also found an effect of physical connectedness in the early visual cortex, suggesting that numerosity segmentation might start from this stage of visual processing (Fornaciai & Park, 2018, 2021).

Hence, the convergence of two separated lines of research investigating the effects of grouping by element connectedness and the ICs formation rules, applied in the context of a numerical task, suggests that numerosity perception could be affected by the hierarchical organization of the raw visual input. Similar types of global biases have been also reported for other hierarchically organized objects, such as (global) letters composed of other smaller (local) letters (Navon, 1977). As a consequence, numerosity perception is a promising field to investigate also the effects of Gestalt grouping cues in visual perception (Roelfsema & Houtkamp, 2011). In addition, the effect of connectedness is in line with recent findings suggesting that object-based attention, as well as location-based attention, may modulate the representation of numerosity (Pomè et al., 2020). Therefore, we pinpoint that our study was carried out to specifically test whether prior works using ICs as connecting lines (Adriano, Rinaldi, et al., 2021; Kirjakovski & Matsumoto, 2016), which used medium numerosities (e.g., 9–15) outside the so-called subitizing range (e.g., less than 5), could be explained by the illusory brightness enhancement confounds in the stimuli. It is worth noting that while some studies using estimation tasks argue against a substantial impact of connectedness in the subitizing range at both behavioral and neural level (Porter et al., 2016; Wurm et al., 2019), others have documented an influence of connectedness over subitizing mechanisms using comparison tasks (He et al., 2015). This suggests that these perceptual manipulations (e.g., connectedness) may depend on several contextual factors such as the experimental task and/or the numerical range used. Indeed, for very large numerosities or dense arrays, the effect of connectedness is weaker or reversed even with comparison tasks (e.g., Anobile et al., 2017; Kirjakovski & Matsumoto, 2016). These apparently diverging findings may be explained by the fact that subitizing and ANS are subserved by different cognitive mechanisms (for a review, see Hyde, 2011).

Visual illusions are thus critical tools to study how visual scene is segmented and to understand the role of low-level features in numerosity processing. The segmentation process carried-out by the boundary contour system, indeed, may also happen at the level of textural differences separating two extended surfaces, and texture qualities may have a key role in preventing the segmentation of the individual items in the texture in a context-dependent manner. For example, when a very large number of dots is presented in the visual field, crowding may occur preventing the correct segmentation of the individual items that may jumble together. Accordingly, Anobile et al. (2014) reported that the Weber fraction was constant for moderate arrays and then decreased with the square root of numerosity for very dense arrays, thus suggesting a texture-density related mechanism for higher numerosities. Recent work suggests that the switch between these systems can be regulated by crowding mechanisms, depending on the visual eccentricity of the stimuli (Anobile et al., 2015; Valsecchi et al., 2013). Hence, for very dense arrays, individual items cannot be segmented, and the single items sometimes may form a regular uniform texture in which individual elements are perceived as part of a larger mesh-like texture (e.g., Kirjakovski & Matsumoto, 2016), which can explain why connectedness is reduced for very large numerosities. As a consequence, textural segmentation process and numerosity processing might be exquisitely context-sensitive mechanisms in line with recent findings (Anobile et al., 2015). That is, a given element at a given location can be part of a variety of larger groupings, depending on the context in which it is presented, and the precise determination even of what acts as an element at a given location can depend on patterns at nearby locations (Grossberg & Mingolla, 1987). Yet it is worth noting that complementary evidence suggests that texture processing and numerosity processing exploit as well different visual mechanisms. For example, Kramer et al. (2011) showed that low-level visual attributes have a little role in numerosity since estimation was robust even when the items were defined by second-order motion (i.e., polarity reversal of the background). Recently, Adriano, Girelli, and Rinaldi (2021) also showed that, for moderate numerosities, texture statistics such as raw spatial frequency alone cannot be a reliable cue to numerosity (see also Railo et al., 2016; Wichmann et al., 2010). However, it is still elusive as to how other continuous features such as convex hull or item space may interact with numerosity in the visual stream. Recently, Chakravarthi and Bertamini (2020) have suggested a unifying theory based on the grouping by item proximity, explaining how numerosity is underestimated when dots are closely spaced, suggesting that close items were segmented together in analogy with the connectedness effect. Future theoretical models should ideally account for the integration of multiple weighted (sometimes redundant/congruent and sometimes nonredundant/incongruent) sensory cues information within the visual scene, including not only continuous (e.g., Gebuis et al., 2016) but also discrete numerosity information (Nys & Content, 2012; see also Cantrell & Smith, 2013) as shown in Stroop-like task, perhaps in a statistical optimal fashion like in cross-modality sensory cue integration (e.g., Alais & Burr, 2004; Ernst & Bülthoff, 2004).

Finally, the strict connection between perceptual organization process and nonsymbolic numerosity perception may explain why individuals affected by clinical conditions such as autism (Aagten-Murphy et al., 2015; Turi et al., 2015) as well as developmental dyscalculia (e.g., Castaldi et al., 2020) may exhibit low abilities in nonsymbolic numerosity tasks. Indeed, it has been shown that perceptual organization may be impaired in autism (Evers et al., 2018) and that autistic children are less affected by physical connectedness of the target items with distractors in attentional task (Evers et al., 2014). Furthermore, people with high autistic-like personality traits are less affected by connectedness of items in numerical arrays of dots grouped by physical lines (Pomè et al., 2021). Although the exact link between atypical perceptual mechanisms and weak numerical estimation skills in autism is far from being understood, their partial dependence is undisputable (Aagten-Murphy et al., 2015; Pomè et al., 2021; Turi et al., 2015). Recently, it has also been shown that dyscalculic children have a larger crowding effect compared with controls (Castaldi et al., 2020). Since a strict link may exist between crowding and grouping/segmentation(Francis et al., 2017) in visual processing, it would be not surprising if further perceptual weakness may unravel and explain the origin of some deficits in dyscalculic subjects, such as impaired ANS representation or worse Weber fraction (precision) in nonsymbolic numerical tasks. Along this line, we suggest that the psychophysical paradigm used in the current work might be particularly suitable to investigate nonverbal numerical competencies not only in typical development but also in autism spectrum disorders, since this method allows to have a fundamental measure of numerical precision (Weber fraction or CoV), as well as of the perceptual organization processing style (e.g., deficit of global perception).

## Conclusions

The current study shows that numerosity perception is not based on continuous cues processing. Our data indeed indicate that increasing illusory connecting lines produces a systematically larger numerosity underestimation effect. This effect is not simply due to physical cues or to brightness confounds (e.g., filling-in process), but rather explained by a unifying process acting independently of contrast polarity, such as the boundary completion process. In sum, our findings suggest that numerosity is computed based on the rapid segmentation of the visual input into coherent segmented objects, reinforcing the idea that numerosity perception might be biased toward a global organization of the visual input.

## Supplementary Information

ESM 1 (DOCX 1355 kb)

## Footnotes

1 Note that we collected overall data from 34 participants, but six of them were discarded from the final sample prior to the analysis because they presented a numerical acuity (e.g., coefficient of variation) that fell above or below the interquartile range (e.g., ±2 *SD*s) of the distribution in one or more conditions, suggesting a very poor or random performance.

2 A total of 27 subjects were tested, but four subjects were discarded from the final sample because they presented a numerical acuity (e.g., coefficient of variation) that fell above or below the interquartile range (e.g., ±2 *SDs*) of the distribution in one or more conditions, suggesting a poor or random performance. Hence analyses were run over a final sample of *N* = 23 subjects.

3 A total of 26 subjects were tested, but two subjects were discarded from the final sample because they presented a numerical acuity (e.g., coefficient of variation) that fell above or below the interquartile range (e.g., ±2 *SD*s) of the distribution in one or more conditions, suggesting a poor or random performance. Hence, analyses were run over a final sample of *N* = 24 subjects.
