# Supplementary material for: Nonsymbolic numerosity in sets with illusory-contours exploits a context-sensitive, but contrast-insensitive, visual boundary formation process
Source: Atten Percept Psychophys. 2021 Oct 17;84(1):205–20. doi: 10.3758/s13414-021-02378-y (PMC8520761; doi:10.3758/s13414-021-02378-y)
Supplement: Supplementary file 1 — (DOCX 1355 kb) [file 13414_2021_2378_MOESM1_ESM.docx]

**Supplementary materials**

**Nonsymbolic numerosity in sets with illusory-contours exploits a context-sensitive, but contrast-insensitive, visual boundary formation process**

Andrea Adriano^1*^, Luca Rinaldi^2, 3 #^, & Luisa Girelli^1,4 #^

*^1^ Department of Psychology, University of Milano-Bicocca (Italy)*

*^2^ Department of Brain and Behavioral Sciences, University of Pavia, Pavia (Italy)*

*^3^ Cognitive Psychology Unit, IRCCS Mondino Foundation, Pavia (Italy)*

*^4^ NeuroMI, Milan Center for Neuroscience, Milano (Italy)*

*****Corresponding author: A. Adriano. Dipartimento di Psicologia, Università degli Studi di Milano-Bicocca, Piazza dell’Ateneo Nuovo 1, Edificio U6, 20126 Milano, Italy.

E-mail: [a.adriano1@campus.unimib.it](mailto:a.adriano1@campus.unimib.it)

^#^ These authors contributed equally to this work.

**Supplementary results: Experiment 1**

We ran two separate Bayesian repeated-measures ANOVAs (2 × 3), on the PSE and CoV, respectively, with the number of ICs and the inducers color as independent variables. From an inspection Table S1 for the PSE, the model that most outperforms the null model is the one with the main effect of ICs only, which received strong evidence in favor of the alternative hypothesis (*BF*_10_ = 66.7). Furthermore, adding the main factor of color of inducers (*BF*_10_ = 21.2) or the interaction (*BF*_10_ = 3.95) makes the model less competitive. We also compared the strength of the Bayes factor against the null model for the models that exclude or include the critical interaction term. The evidence *against* including the interaction is roughly a factor of six, compared with the model with the main factors. This can be obtained as 21.2 */* 3.95 ≈ 5*.*36. Thus, the data are almost 6 times more likely under the two main effects model than under the full model (i.e., the one including also the interaction). Similarly, the data are 17 times more likely under the model with the main effect of ICs only, compared to the full model (66.7/3.95 ≈ 16.88). Finally, the main effect of color of inducers received substantial support in favor of the null hypothesis (*BF*_10_ = 0.311). In sum, the Bayesian ANOVA for the PSE reveals that the data provide very strong support for the main effect of ICs, but good evidence against the color of inducers. The data also provide good evidence against including the interaction term.

The analysis of CoV revealed that the full model, containing the main effects and the interaction, received strong evidence in favor of the alternative hypothesis (*BF*_10_ = 0.031) compared with all the other simple models (see Table S2).

| **Table S1** Bayesian ANOVA on the PSE  **Model comparison** | | | | | | | | | | | |
| --- | --- | --- | --- | --- | --- | --- | --- | --- | --- | --- | --- |
| **Models** | | **P(M)** | | **P(M\|data)** | | **BF _M_** | | **BF _10_** | | **error %** | |
| Null model (incl. subject) |  | 0.200 |  | 0.011 |  | 0.043 |  | 1.000 |  |  |  |
| ICs |  | 0.200 |  | 0.716 |  | 10.074 |  | 66.721 |  | 0.760 |  |
| Inducer color + ICs |  | 0.200 |  | 0.228 |  | 1.180 |  | 21.232 |  | 1.699 |  |
| Inducer color + ICs + Inducer color  ×  ICs |  | 0.200 |  | 0.042 |  | 0.177 |  | 3.950 |  | 4.411 |  |
| Inducer color |  | 0.200 |  | 0.003 |  | 0.013 |  | 0.311 |  | 3.751 |  |
|  | | | | | | | | | | | |
| *Note.* All models include subject. | | | | | | | | | | | |

| **Table S2** Bayesian ANOVA on the CoV  **Model comparison** | | | | | | | | | | | |
| --- | --- | --- | --- | --- | --- | --- | --- | --- | --- | --- | --- |
| **Models** | | **P(M)** | | **P(M\|data)** | | **BF _M_** | | **BF _10_** | | **error %** | |
| Null model (incl. subject) |  | 0.200 |  | 0.543 |  | 4.756 |  | 1.000 |  |  |  |
| ICs |  | 0.200 |  | 0.277 |  | 1.529 |  | 0.509 |  | 1.852 |  |
| Inducers color |  | 0.200 |  | 0.109 |  | 0.487 |  | 0.200 |  | 2.033 |  |
| Inducers color + ICs |  | 0.200 |  | 0.055 |  | 0.233 |  | 0.101 |  | 1.966 |  |
| Inducers color + ICs + Inducers color  ×  ICs |  | 0.200 |  | 0.017 |  | 0.068 |  | 0.031 |  | 2.132 |  |
|  | | | | | | | | | | | |
| *Note.* All models include subject. | | | | | | | | | | | |


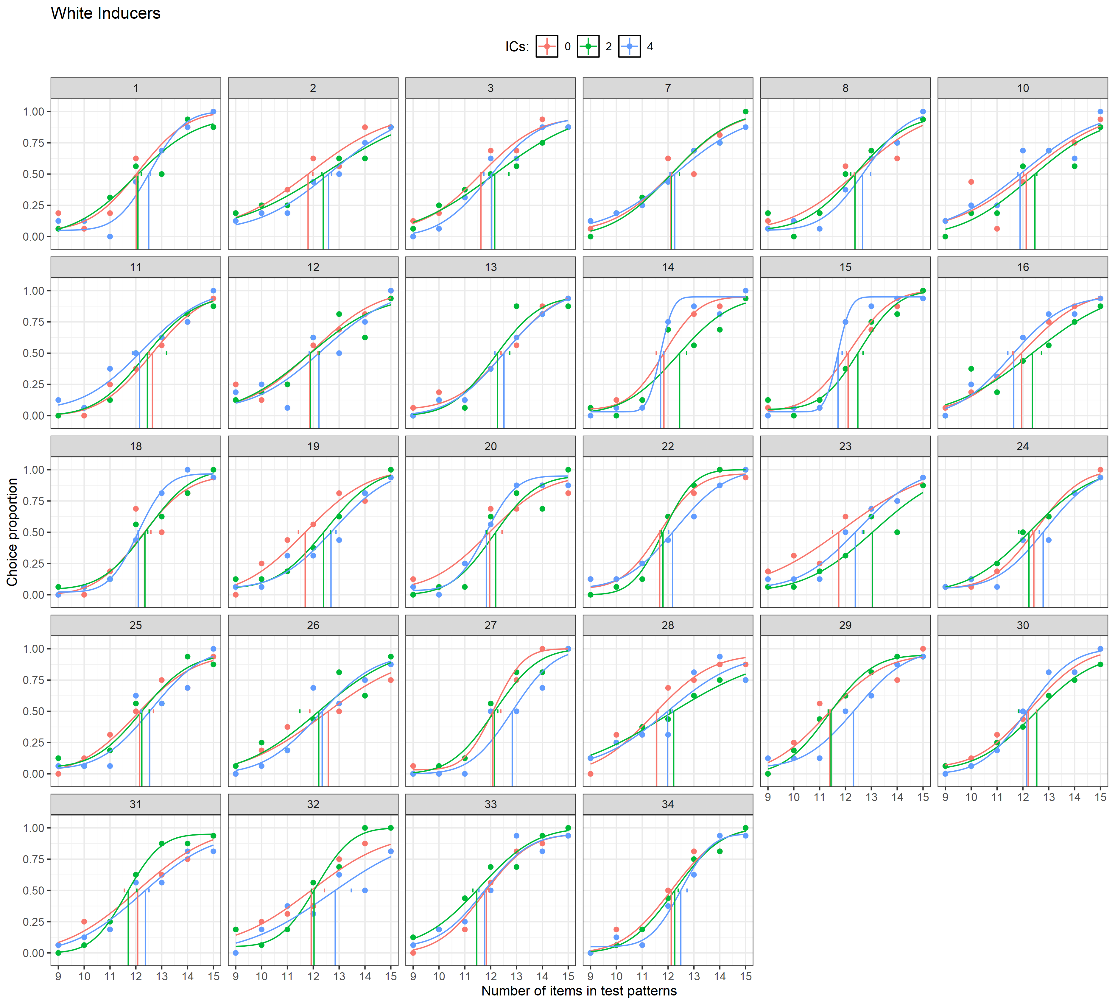


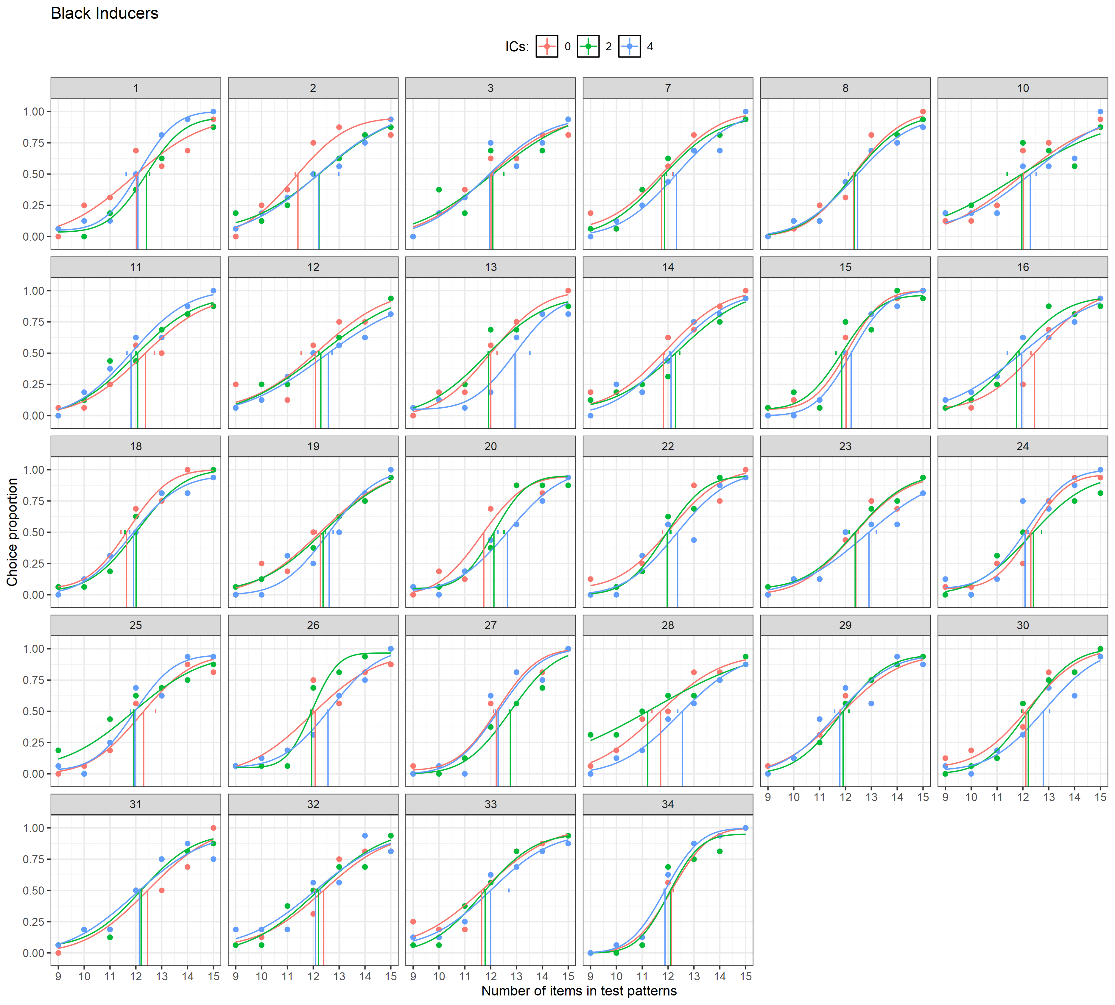


**Fig. S1** Individual psychometric functions for Experiment 1


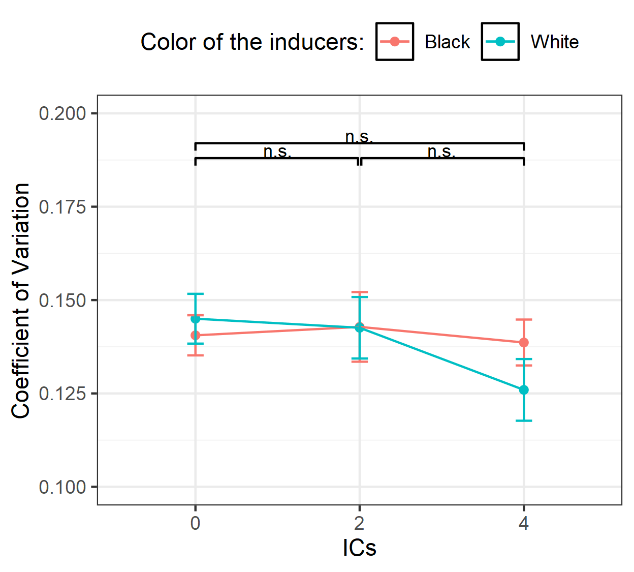


**Fig. S2** Coefficient of variation as a function of each condition. The error bars represent ±1 *SEM*

**Supplementary results: Experiment 2**

As in the Experiment 1, we ran two separate one-way Bayesian repeated-measures ANOVAs respectively on the PSE and CoV, with the number of ICs as independent variable. The analysis of PSE (Table S3) revealed that the factor number of ICs received overwhelming evidence in favor of the alternative hypothesis (*BF*_10_ = 707.1). Furthermore, the analysis of the CoV (Table S4) suggested that the factor number of ICs received anecdotal evidence in favor of the null hypothesis (*BF*_10_ = 0.399).

| **Table S3** Bayesian ANOVA on the PSE  **Model comparison** | | | | | | | | | | | |
| --- | --- | --- | --- | --- | --- | --- | --- | --- | --- | --- | --- |
| **Models** | | **P(M)** | | **P(M\|data)** | | **BF _M_** | | **BF _10_** | | **error %** | |
| Null model (incl. subject) |  | 0.500 |  | 0.001 |  | 0.001 |  | 1.000 |  |  |  |
| ICs |  | 0.500 |  | 0.999 |  | 707.102 |  | 707.102 |  | 1.674 |  |
|  | | | | | | | | | | | |
| *Note.* All models include subject. | | | | | | | | | | | |

| **Table S4** Bayesian ANOVA on the CoV  **Model comparison** | | | | | | | | | | | |
| --- | --- | --- | --- | --- | --- | --- | --- | --- | --- | --- | --- |
| **Models** | | **P(M)** | | **P(M\|data)** | | **BF _M_** | | **BF _10_** | | **error %** | |
| Null model (incl. subject) |  | 0.500 |  | 0.715 |  | 2.504 |  | 1.000 |  |  |  |
| ICs |  | 0.500 |  | 0.285 |  | 0.399 |  | 0.399 |  | 1.271 |  |
|  | | | | | | | | | | | |
| *Note.* All models include subject. | | | | | | | | | | | |


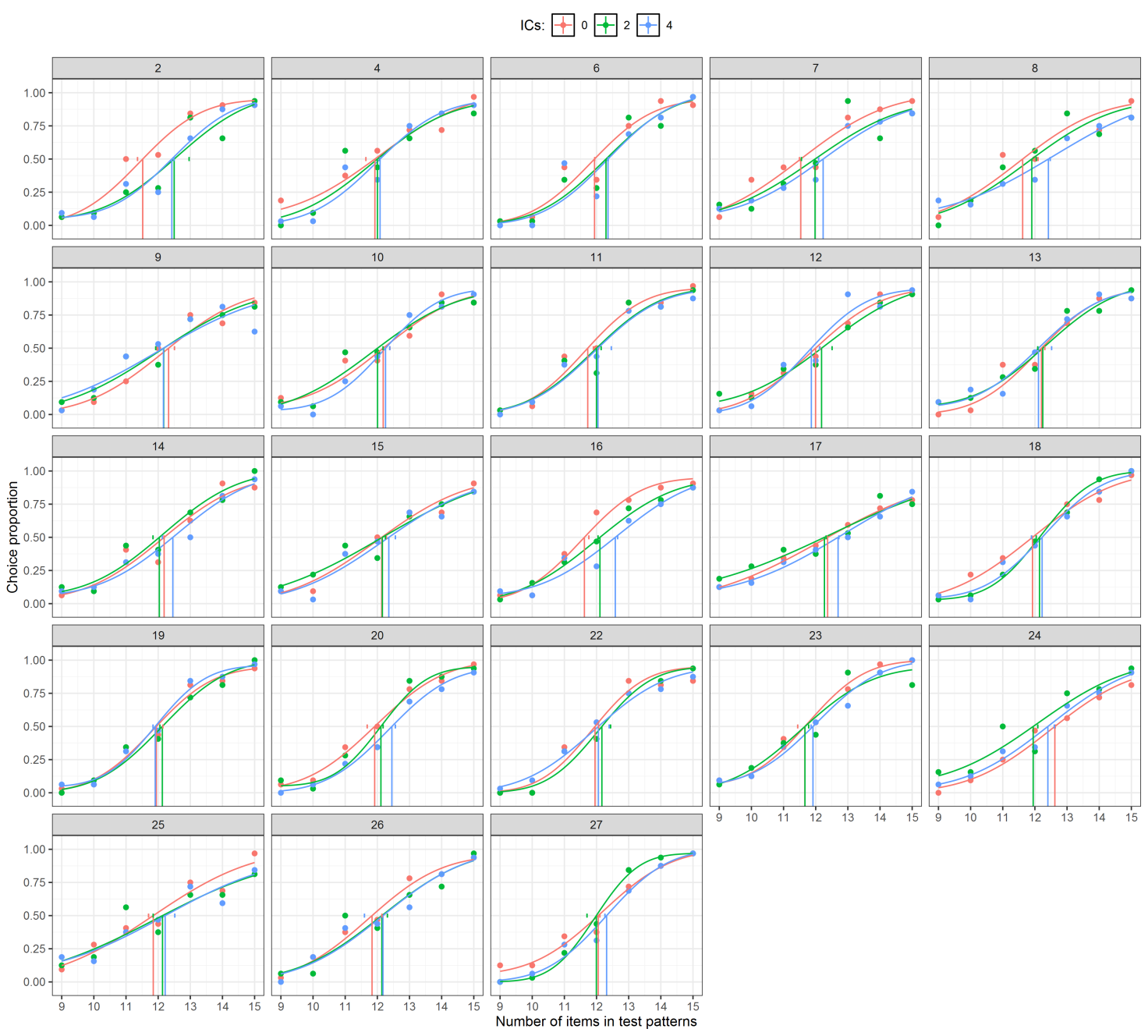


**Fig. S3** Individual psychometric functions for Experiment 2


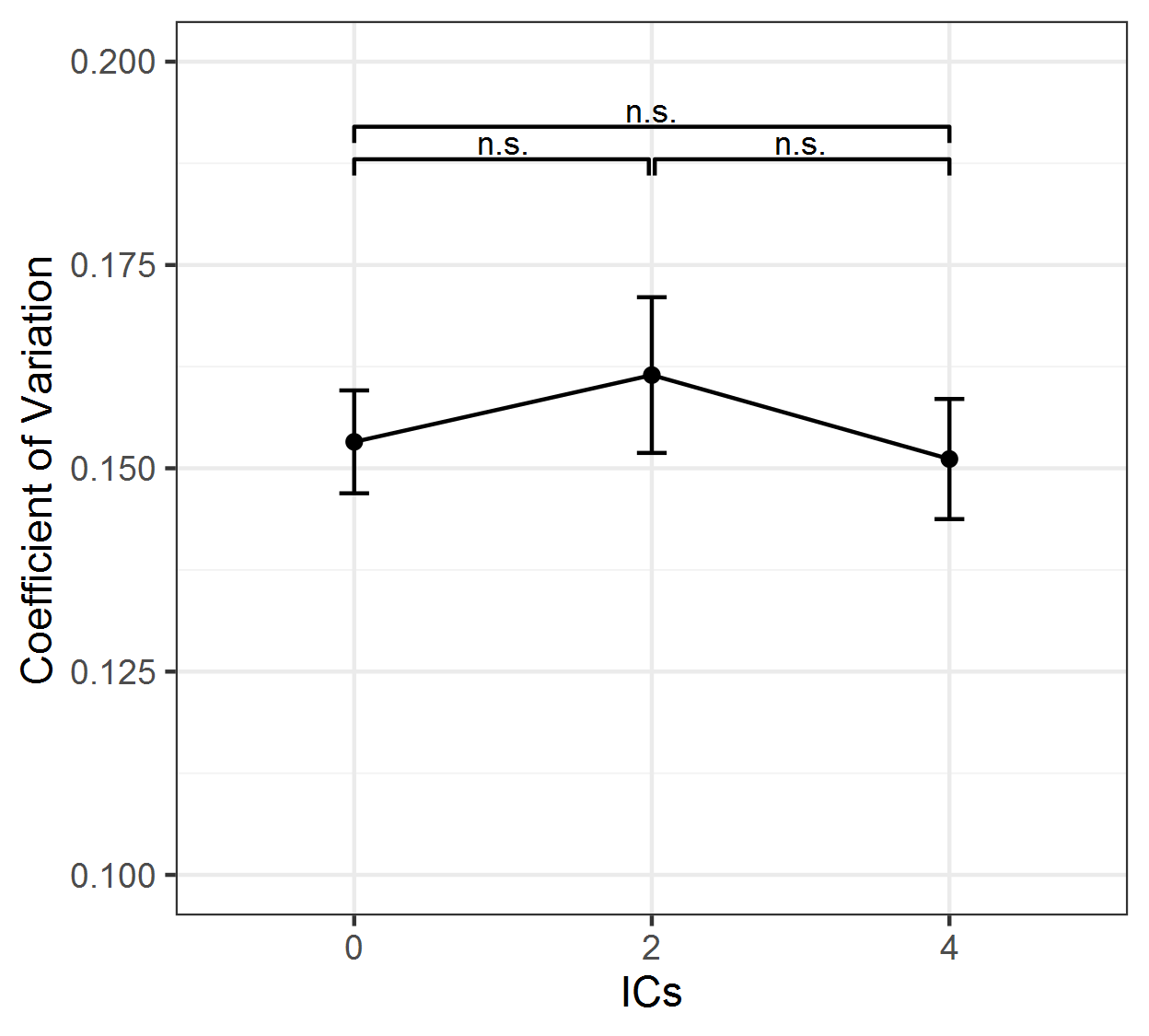


**Fig. S4** Coefficient of variation as a function of each condition. The error bars represent ±1 *SEM*

**Supplementary results: Experiment 3**

As in the previous experiments, we ran two separate one-way Bayesian repeated-measures ANOVAs, on the PSE and CoV, respectively, with the number of ICs as independent variable, as in the second experiment. The analysis of PSE (Table S5) revealed that the factor number of ICs received only anecdotal evidence in favor of the null hypothesis (*BF*_10_ = 0.41). Finally, the analysis of the CoV (Table S6) suggested that the factor number of ICs received substantial evidence in favor of the null hypothesis (*BF*_10_ = 0.132).

| **Table S5** Bayesian ANOVA on the PSE  **Model comparison** | | | | | | | | | | | |
| --- | --- | --- | --- | --- | --- | --- | --- | --- | --- | --- | --- |
| **Models** | | **P(M)** | | **P(M\|data)** | | **BF _M_** | | **BF _10_** | | **error %** | |
| Null model (incl. subject) |  | 0.500 |  | 0.707 |  | 2.409 |  | 1.000 |  |  |  |
| ICs |  | 0.500 |  | 0.293 |  | 0.415 |  | 0.415 |  | 0.504 |  |
|  | | | | | | | | | | | |
| *Note.* All models include subject. | | | | | | | | | | | |

| **Table S6** Bayesian ANOVA on the CoV  **Model comparison** | | | | | | | | | | | |
| --- | --- | --- | --- | --- | --- | --- | --- | --- | --- | --- | --- |
| **Models** | | **P(M)** | | **P(M\|data)** | | **BF _M_** | | **BF _10_** | | **error %** | |
| Null model (incl. subject) |  | 0.500 |  | 0.883 |  | 7.556 |  | 1.000 |  |  |  |
| ICs |  | 0.500 |  | 0.117 |  | 0.132 |  | 0.132 |  | 1.045 |  |
|  | | | | | | | | | | | |
| *Note.* All models include subject. | | | | | | | | | | | |


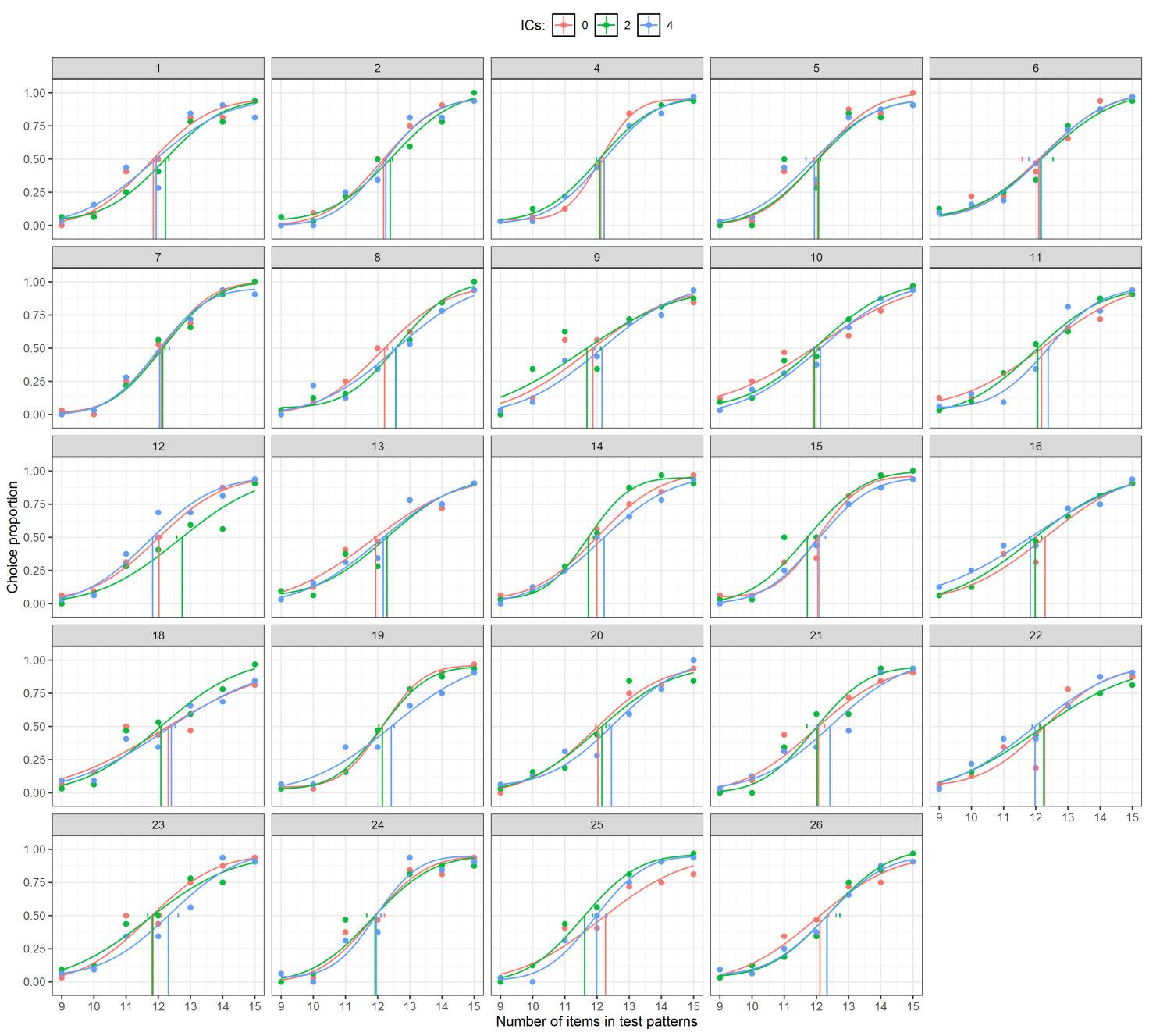


**Fig. S5** Individual psychometric functions for Experiment 3


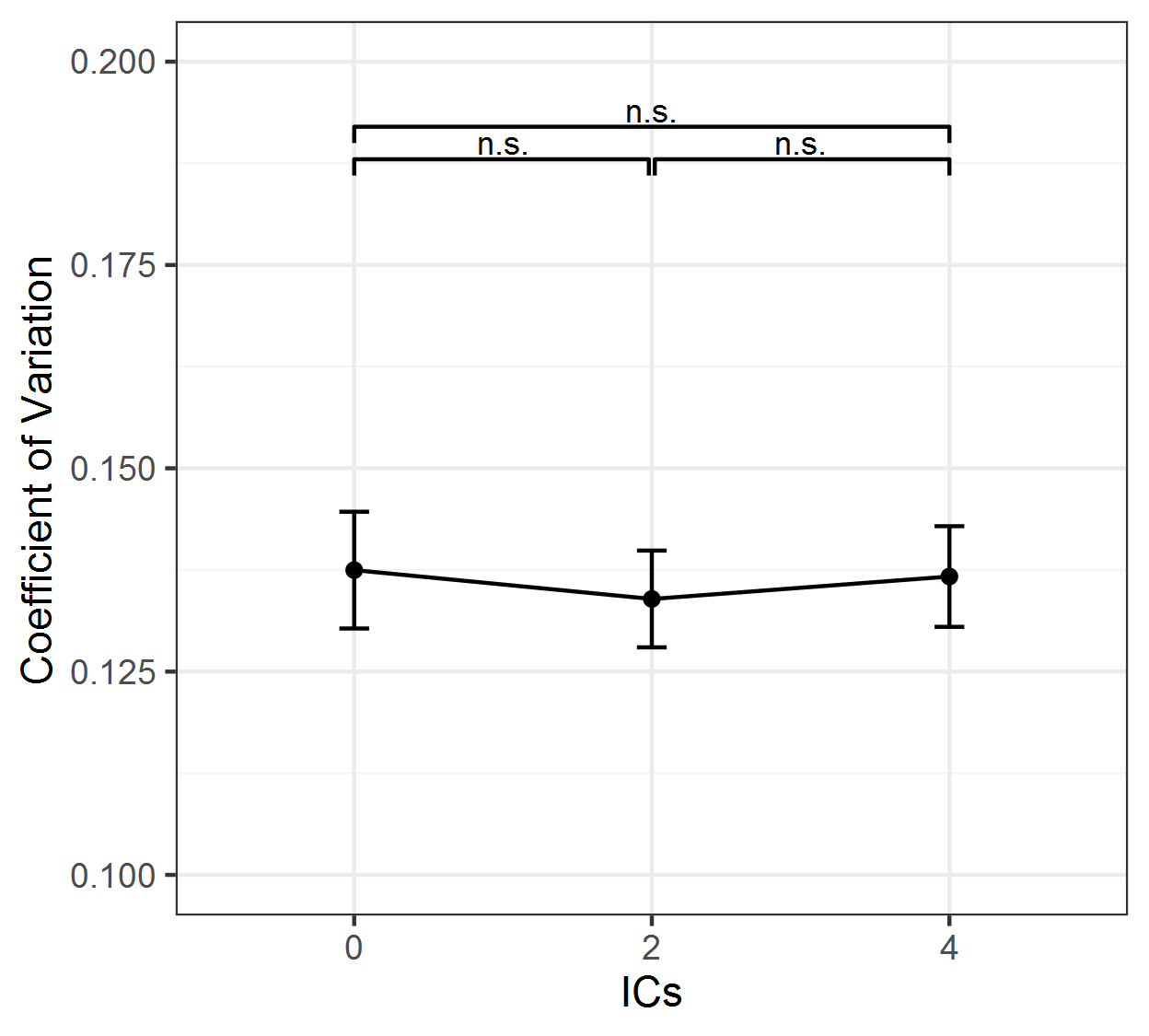


**Fig. S6** Coefficient of variation as a function of each condition. The error bars represent ±1 *SEM*
